# The usefulness of school-based syndromic surveillance for detecting malaria epidemics: experiences from a pilot project in Ethiopia

**DOI:** 10.1186/s12889-015-2680-7

**Published:** 2016-01-09

**Authors:** Ruth A. Ashton, Takele Kefyalew, Esey Batisso, Tessema Awano, Zelalem Kebede, Gezahegn Tesfaye, Tamiru Mesele, Sheleme Chibsa, Richard Reithinger, Simon J. Brooker

**Affiliations:** 1 Malaria Consortium, London, UK; 2 Faculty of Infectious and Tropical Diseases, London School of Hygiene & Tropical Medicine, London, UK; 3 Malaria Consortium Ethiopia, Addis Ababa, Ethiopia; 4 Malaria Consortium Southern Nations, Nationalities and People’s Regional State sub-office, Hawassa, Ethiopia; 5 Southern Nations, Nationalities and People’s Regional State Health Bureau, Hawassa, Ethiopia; 6 President’s Malaria Initiative, U.S. Agency for International Development, Addis Ababa, Ethiopia; 7 RTI International, Washington, DC USA

**Keywords:** Malaria, Syndromic surveillance, Schools, Epidemics, Absenteeism

## Abstract

**Background:**

Syndromic surveillance is a supplementary approach to routine surveillance, using pre-diagnostic and non-clinical surrogate data to identify possible infectious disease outbreaks. To date, syndromic surveillance has primarily been used in high-income countries for diseases such as influenza -- however, the approach may also be relevant to resource-poor settings. This study investigated the potential for monitoring school absenteeism and febrile illness, as part of a school-based surveillance system to identify localised malaria epidemics in Ethiopia.

**Methods:**

Repeated cross-sectional school- and community-based surveys were conducted in six epidemic-prone districts in southern Ethiopia during the 2012 minor malaria transmission season to characterise prospective surrogate and syndromic indicators of malaria burden. Changes in these indicators over the transmission season were compared to standard indicators of malaria (clinical and confirmed cases) at proximal health facilities. Subsequently, two pilot surveillance systems were implemented, each at ten sites throughout the peak transmission season. Indicators piloted were school attendance recorded by teachers, or child-reported recent absenteeism from school and reported febrile illness.

**Results:**

Lack of seasonal increase in malaria burden limited the ability to evaluate sensitivity of the piloted syndromic surveillance systems compared to existing surveillance at health facilities. Weekly absenteeism was easily calculated by school staff using existing attendance registers, while syndromic indicators were more challenging to collect weekly from schoolchildren. In this setting, enrolment of school-aged children was found to be low, at 54 %. Non-enrolment was associated with low household wealth, lack of parental education, household size, and distance from school.

**Conclusions:**

School absenteeism is a plausible simple indicator of unusual health events within a community, such as malaria epidemics, but the sensitivity of an absenteeism-based surveillance system to detect epidemics could not be rigorously evaluated in this study. Further piloting during a demonstrated increase in malaria transmission within a community is recommended.

## Background

Infectious diseases inherently exhibit marked spatial and temporal trends [1–4], which can manifest as epidemics. Epidemics are broadly defined as unusual increases in the burden of illness, that are clearly in excess of normal expectancy [5]. Definitions of “normal” burden vary, but upper and lower limits of normality are often defined as being two standard deviations around the mean number of cases for a facility in a defined time period, after excluding previous epidemic periods [6]. Comprehensive surveillance systems are crucial to enable the timely identification of unusual increases in disease incidence, to minimise onward spread through early detection and treatment of affected individuals and to effectively target control measures. Detection of individuals with an epidemic-prone infectious disease is typically based on clinical or biological diagnosis. Another approach to detecting individuals with disease is the use of pre-diagnostic or surrogate indicators, described as syndromic surveillance.

School absenteeism is one such surrogate indicator. School absenteeism is appealing as an indicator for syndromic surveillance since it utilises an existing and well-established source of data in the form of daily school attendance registers, and has a fine temporal resolution. The primary application of school absenteeism for syndromic surveillance has been for detection of influenza epidemics in high-income countries [7–10], where school enrolment and attendance is generally high and thus schoolchildren are expected to be representative of the wider population. Prior studies using school absenteeism as an indicator of infectious disease outbreaks in resource-poor settings are few. In China, a web-based surveillance system incorporated primary school absenteeism, health facility syndromic data and pharmacy medication sales [11, 12]. In Cambodia, an approach using school absenteeism data submitted by short message service (SMS) to a central server was found to be feasible and acceptable [13].

School absenteeism is a recognised consequence of malaria epidemics that occur in the highlands of East Africa [14], where malaria transmission varies in time and space. Ethiopia has seen some of the largest malaria epidemics in the region with very high case fatality rates [15]. While no severe epidemics have been observed in Ethiopia since 2004, and more recent district-level outbreaks have been successfully contained, both small and large scale epidemics continue to be a significant health threat [16]. As per WHO guidance [17], routine detection of epidemics in Ethiopia is based on health facilities plotting weekly total confirmed malaria cases against a threshold calculated using five years’ historical data [18], but this method fails to account for temporal variations in peak transmission [19]. Furthermore, delays in data reporting and incomplete or inaccurate data limit application of the health facility-based epidemic detection system [20–22]. While these limitations persist, alternative tools or strategies that can bridge these gaps and facilitate early identification of epidemics are needed. The need for community-based approaches to malaria surveillance and response, tailored to the local setting, have also been highlighted as a priority in achieving malaria elimination [23]. The dramatic increase in primary school enrolment in Ethiopia and other African countries [24], suggests that school-level malariometric indicators such as prevalence by rapid diagnostic test (RDT) are increasingly representative of the wider population [25]. Febrile illness may be an additional useful indicator for identification of malaria epidemics. While a large proportion of infections in low transmission settings such as Ethiopia are of low parasite density and asymptomatic [26–28], it is hypothesised that during an epidemic symptomatic illness would increase, due to the substantial increase in the total number of *Plasmodium* infections.

The current study was designed to explore the usefulness of a syndromic surveillance approach to identify unusual increases in malaria at community-level in a low-income country, Ethiopia. The specific objectives were to: (i) assess the feasibility of different approaches to routinely collect and analyse indicators from rural primary schools in areas at risk of malaria epidemics; (ii) assess the correlation between changes in these surrogate and syndromic indicators with parasitological indicators of malaria burden within the wider population through cross-sectional surveys and passive health facility case detection; and (iii) specifically investigate the reasons for school absenteeism in this environment and, therefore, the potential of absenteeism as a surrogate surveillance indicator.

### Overview of syndromic surveillance approaches

Syndromic surveillance refers to the use of pre-diagnostic health indicators to allow timely detection and investigation of potential infectious disease outbreaks [29] as a supplementary approach to routine public health surveillance, by enabling early identification of clusters of illness before confirmatory data are available. In addition to use of clinical (syndrome) data, syndromic surveillance can be expanded to include surrogate non-clinical data indicating early illness, through mining of available data to track changes in infectious diseases in the population. Surrogate data sources include prescription and over-the-counter drug sales, internet search terms and social media [30–36] and school absenteeism [7–10, 37]. The latter is an alternative indicator of population health that has been applied to monitor influenza outbreaks in high-income countries, but yet to be fully explored as an approach for infectious disease surveillance in resource poor settings. Syndromic surveillance systems piloted in resource poor settings have to date used clinical signs among patients attending health facilities as their indicators [38–42], but two examples of school absenteeism being used as early warning of outbreaks of respiratory and gastrointestinal diseases are available from Cambodia and rural China [11, 13, 43]. The key surveillance studies using school absenteeism for outbreak detection, as well as classic applications of syndromic surveillance utilising data on clinical morbidities are presented in Table 1, to demonstrate the various settings, indicators, temporal resolution and complexity of these syndromic surveillance systems.

**Table 1:** Selected syndromic surveillance systems reported in the literature: the setting, target diseases, indicators, system complexity and outcomes of their application. Reported studies are those which use school absenteeism as a key indicator, or systems applied in resource-limited settings for epidemic prone diseases including malaria

Setting	Target disease(s)	Indicators	Reporting frequency	Complexity of system	Surveillance system findings	Ref
Canada	H1N1 influenza	Elementary and high school absenteeism due to influenza-like illness exceeding the defined threshold of 10 % of total enrolment	Daily analysis of absenteeism, reporting if exceed threshold	Low – schools report data when indicator exceeds the threshold	Absenteeism was well correlated with hospitalisation rates for school age children and PCR positive tests for influenza. Peak absenteeism preceded peaks in hospitalisations by one week	[7]
United Kingdom	H1N1 influenza	School absenteeism in primary and secondary schools, comparing against telephone health hotline, general practitioner sentinel network & confirmed influenza data	Weekly mean percentage absenteeism	Low – collation of school % absenteeism data	Weekly school absenteeism peaked concomitantly with existing influenza alert systems, and would not have identified pandemic influenza earlier than other systems. Daily attendance data may have improved timeliness	[8]
Japan	Influenza	School influenza-related absenteeism, where child absent with confirmed diagnosis from physician	Daily school influenza-related absenteeism rate	Low – daily attendance routinely recorded and absent children require doctor’s note	School influenza-related illness can be used to predict outbreaks and determine when a school should close to limit ongoing spread. Thresholds for influenza-related absenteeism proposed.	[9]
China (rural)	Respiratory infections, gastroenteritis	Symptoms reported at health clinics, over-the-counter drug sales at pharmacies and primary school absenteeism	Daily input to web-based system	High – collation and analysis of data at central level	Labour-intensive data entry to electronic system. Presentation of six months’ pilot data, no validation of data from surveillance system against other sources	[11, 43]
Madagascar	Malaria, influenza, dengue, diarrhoeal disease	Malaria case confirmed by RDT, fever & respiratory symptoms, fever & 2 possible dengue symptoms, diarrhoea.	Daily report by encrypted SMS. Weekly summary paper report.	Moderate – SMS reports entered to database. Temporal & spatial analysis by syndrome	Ten cases of fever clusters occurred which weren’t detected by the traditional surveillance system. Five outbreaks identified – two dengue, two influenza and one malaria.	[42]
French Guiana	Dengue	Dengue index: percentage of patients attending the emergency department who had thrombocytopenia but were negative for Plasmodium infection	Weekly generation of indicators	Low – plotting of simple indicators on weekly basis, minimal analysis	Dengue index was specific – increasing during what was confirmed to be a dengue epidemic, but showing no strong increase during two respiratory infection epidemics. Total emergency department attendance with thrombocytopenia but malaria negative was also a specific indicator.	[38]
Pacific island countries and territories	Measles, dengue, rubella, meningitis, leptospirosis, gastroenteritis, influenza, typhoid, malaria	Hospitals report total cases for four syndromes: acute fever & rash, diarrhoea, influenza-like illness, prolonged fever	Weekly reporting of data to national level	Moderate – data reported from national to WHO regional level for analysis	The system successfully identified an outbreak of diarrhoeal disease linked to breakdown of water disinfection, and two outbreaks of influenza. The system alert was timely and allowed fast implementation of control measures	[39]
India	Cholera, dysentery, malaria, measles, meningitis, typhoid fever, and 8 others	Suspected cases (clinical diagnosis) of target diseases from public and private health facilities, except malaria, where slide-confirmation required for reporting	As clinical cases identified (daily), using pre-formatted post cards with postage pre-paid	Low – doctors report cases on simple form to central level. Minimal analysis.	Several outbreaks were detected early and interventions applied, the most notable was cholera. Leptospirosis and acute dysentery also commonly reported. Monthly summary of reported diseases distributed to participating facilities for feedback and updates on the surveillance system.	[41]
Cambodia	Respiratory and diarrhoeal diseases	School absenteeism (aggregated daily by schools), compared against overall health facility attendance	Daily SMS report of school absenteeism due to illness, collated at weekly level for analysis	Low - daily data reported by schools to central level, compared against all cause health centre attendance	Illness-specific absenteeism identified two peaks in incidence of illness. Absenteeism data preceded peaks in health centre attendance by 0.5 weeks on average. Cross correlation analysis indicated moderate correlations between illness specific absenteeism and reference data.	[13]
Papua New Guinea	Influenza, cholera, typhoid, malaria, poliomyelitis, meningitis, measles, dengue	Syndromes relating to target diseases identified in patients presenting to health facilities.	Weekly report by mobile phone, transcription to database	Low – health facilities submit data for analysis at provincial/national level, and automatic generation of feedback reports	System was more sensitive than the reference system for measles, but low sensitivity for malaria, due to poor case definition. Data were more timely than the reference system (mean 2.4 weeks compared to 12 weeks lag)	[54]

## Methods

### Study design

**Fig. 1:**
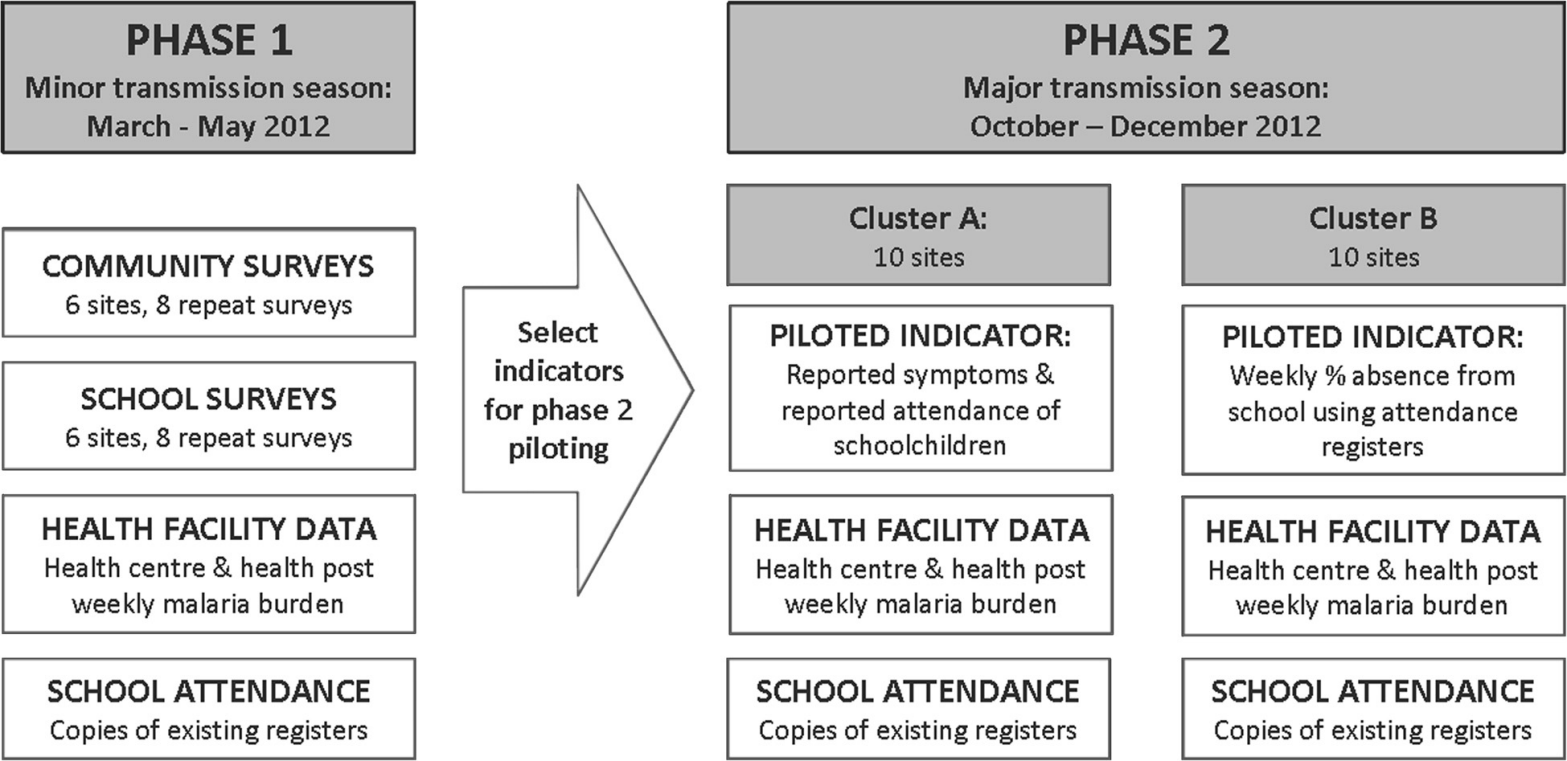
Study design diagram indicating activities conducted during Phase 1 (school- and community-based surveys) and Phase 2 (piloting of two school-based syndromic surveillance systems). Heath facility and school attendance data were collected throughout Phases 1 and 2

The study was conducted in Southern Nations, Nationalities and People’s Regional State (SNNPRS), Ethiopia, and divided into two phases. Phase 1 comprised cross-sectional school- and household-based surveys at six sites during the minor malaria transmission season (March–May 2012), collection of routinely recorded school attendance data, and weekly summary of clinical and confirmed malaria cases at health facilities serving the study sites. Phase 1 activities aimed to define appropriate indicators for further piloting as part of a syndromic surveillance approach. Two school-based syndromic surveillance approaches were implemented as Phase 2 of the study during the peak transmission season (October 2012 to January 2013), each system being piloted at 10 sites in SNNPRS (Fig. 1).

### Cross-sectional surveys at school- and community-level (Phase 1)

**Fig. 2:**
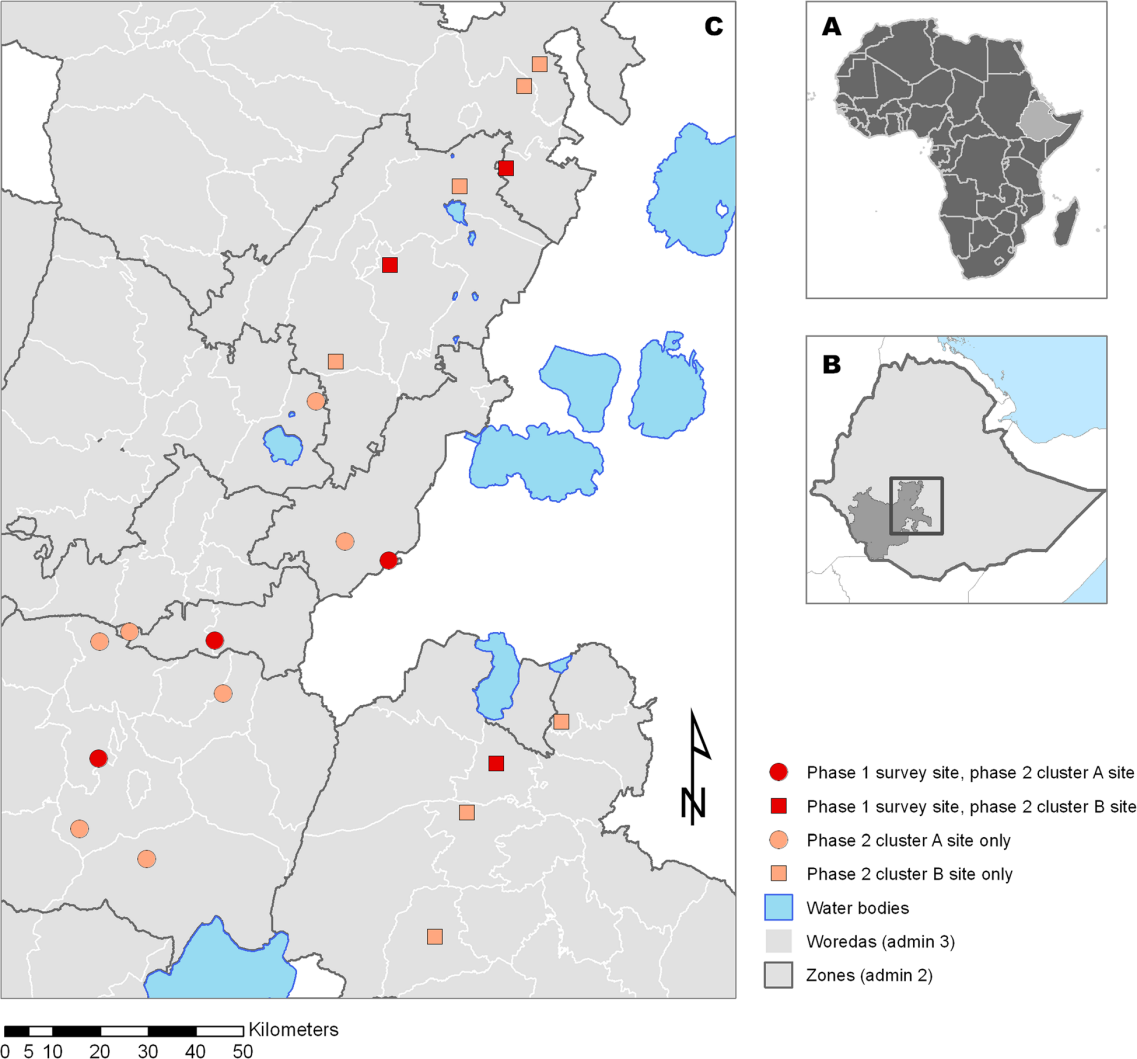
Locator maps of Ethiopia (**a**) and SNNPRS (**b**), with a map of study kebele location (**c**) Six sites which were included in the Phase 1 school and community surveys as well as Phase 2 pilot are indicated by red markers, while the remaining 14 sites participating in Phase 2 pilots only are indicated by orange markers. Assignment to cluster A (symptom questionnaire) during Phase 2 is indicated by circular markers, assignment to cluster B (absenteeism estimated from attendance registers) is indicated by square markers

Six woredas (districts) were chosen purposively from those designated as “hotspot” woredas by the SNPPRS Regional Health Bureau, indicating epidemic risk and higher malaria burden relative to other woredas (Fig. 2). All sites were located at 1850–2000 metres altitude, along the Rift Valley. Sites included flatlands with savannah-type vegetation, where the main crops are teff and pepper, and highland slopes with abundant vegetation where the main cash crop is coffee. Coverage of health facilities in the areas was in line with the national policy for a “primary health care unit” of one health centre and five satellite health posts for each 25,000 people. In practice, each kebele (municipality) tends to have at least one health post and each woreda at least one health centre [44].

From each woreda, one kebele was purposively chosen as the study site, with inclusion criteria being: government primary school with at least 100 children attending; a health post; <200 metres altitude range; and, accessibility during the rainy season. “School-aged” in the current study was defined as age seven to 16 years, since national policy is for children to enrol in school at seven years of age [45].

Eight repeat school and community surveys were conducted at approximately ten-day intervals from March to May 2012. Due to lack of prior data describing likely range of syndromic indicators of interest, no sample size calculation was completed, but sample size was maximised within logistical capacity.

The community survey protocol followed Malaria Indicator Survey procedures, mapping and randomly selecting 25 households per survey site [46], but with the primary sampling unit defined as primary school catchment area. Random selection of participating households was repeated for each of the eight survey iterations. A questionnaire was completed for each household and all individuals at the household were invited to provide a single finger prick blood sample for multi-species RDT (CareStart HRP2/panLDH, AccessBio, USA) and blood film. School surveys followed a standard methodology previously used in Ethiopia and Kenya [47, 48], with random selection of children was repeated at each survey iteration. Each child completed a questionnaire and was requested to provide a single finger-prick blood sample for multi-species RDT and blood film.

Attendance register data were extracted to calculate weekly absenteeism by class, defined as total child-days absence recorded, divided by the product of total children enrolled and number of days that attendance was recorded by the teacher. In addition, weekly malaria case data were collected from health centres and health posts serving the six study sites. Indicators included clinical and confirmed malaria cases and number tested by microscopy (at health centres) or RDT (at health posts).

### Assigning sites to the two pilot systems (Phase 2)

Design of two surveillance systems was informed by Phase 1 findings, including feasibility of collecting various indicators at school-level, and the correlation between the indicator and number of malaria cases reported at health facilities. Indicators selected for piloting during Phase 2 were: weekly school absenteeism measured by attendance register, and the proportion of school-attending children reporting recent fever, recent absence from school or absence from school due to illness.

The two surveillance systems were piloted during the second school semester, from early October 2012 to the first week of January 2013 when the semester ended. All sites included in Phase 1 were retained for Phase 2. A further 14 sites (from 14 woredas) were purposively chosen using the same inclusion criteria as for Phase 1 sites. Woredas were assigned to participate in either the cluster A or B pilot without randomisation, according to location of woreda within higher administrative unit (zones) (Fig. 2). This approach was used to reduce potential confusion between the two pilot methodologies by any zone health office staff supporting implementation. A one-day training event was held for three individuals from each site (the school director, a teacher and a representative from the woreda health office) on the relevant pilot methodology.

### Piloted surveillance system methodology (Phase 2)

Cluster A sites used symptom questionnaires to investigate schoolchildren’s reported recent fever, absenteeism due to illness and absenteeism for any reason. The symptom questionnaire was completed by a teacher every Monday, immediately after the attendance register. Each child was called in turn to the teacher’s desk to respond to the questionnaire. The symptom questionnaire was restricted to grades two to four inclusive, since higher grades were unavailable due to exam preparations. Interviews were rotated weekly between grades to minimise disruption to normal teaching. The questionnaire included 10 symptoms (e.g. headache, cough, stomach ache), which were primarily included to mask fever as the symptom of interest; however, the additional symptoms could be of interest if the system was adopted with a remit including other epidemic-prone infectious diseases.

Cluster B sites were simply requested to use data recorded in their usual attendance registers to complete a weekly summary across all grades of the proportion absent, with total children enrolled multiplied by number of days attendance recorded as the denominator. At the end of the pilot period, copies of attendance registers were collected for validation purposes from a convenience sample of schools. Weekly health facility malaria data were also collected from 20 health centres and 20 health posts serving the Phase 2 study populations.

### Data entry and analysis

Questionnaire data from Phase 1 surveys were entered into a customised Microsoft Access 2007 database with consistency and range checks. Microscopy results, health facility data and absenteeism extracted from attendance registers were entered into Microsoft Excel. Data were merged in Stata 12.0 (Stata Cooperation, College Station, Texas USA). Household coordinates for Phase 1 sites were imported into ArcMap 10.0 (Environmental Systems Research Institute Inc., Redlands, California USA) for display and calculation of Euclidean distance between household and school (at approximately 100 m resolution). Phase 2 data were entered into Excel spreadsheets and exported to Stata 12.0 for merging and analysis.

### Analysis of Phase 1 data

The primary aim of Phase 1 analysis was to identify indicators which could be further piloted in Phase 2 school-based surveillance system, with secondary aims to determine the representativeness of the school-enrolled population compared to the wider community, and assess reported reasons for short-term absence from school.

A wealth index was created using principal component analysis [49], at household level for community survey data and individual level for school survey data. Indicators associated with school enrolment reported during community surveys were assessed using binomial extension to generalised linear modelling at household level, with standard error estimates adjusted for clustering by study site. Mixed effects logistic regression was used to develop multivariate multilevel models describing risks of non-enrolment in school, with household-level and site-level random effects. A backward step-wise method was used to exclude the fixed effects in order of least significance: a likelihood ratio test was used to re-test excluded variables for inclusion in the final model.

### Analysis of Phase 2 data

Analysis of Phase 2 data focussed upon describing the characteristics of indicators collected at schools during the pilot. Box plots and logistic regression were used to describe absenteeism by grade over the study period. To explore dropout levels in these populations, mean absenteeism was evaluated by time. Accuracy of weekly summary absenteeism calculated by cluster B sites was determined by comparing teacher-generated summaries against the original registers.

### Ethical considerations

Approval for this study was granted by the London School of Hygiene & Tropical Medicine ethical committee (6003) and the SNNPRS Health Research Ethics Review committee (P026-19/6157). Written, informed consent for participation of children in school surveys was collected from parents. Parents were free to withdraw consent at any time by informing the school director. Children were informed of the study procedures and their right to withdraw prior to random selection, and gave assent to take part. The head of household provided written consent for inclusion of household members in community survey, and verbal assent was sought from all household members before collection of blood samples.

A community health extension worker was present throughout school and community surveys. Participants with positive RDT were provided with treatment according to national guidelines: chloroquine for *P. vivax* and artemether-lumefantrine for *P. falciparum* or mixed infections.

## Results

### Population participating in Phase 1 school- and community-based surveys

RDT results were available from 4117 individuals participating in community surveys, aged from two months to 101 years (median 14 years). A total of 5189 school aged children participated in the school survey, with RDT results available from 5145 children (RDT data missing for 44 (0.8 %) of school children).

The prevalence of *Plasmodium* infection by RDT across all sites and survey iterations was 2.0 % (range across site and survey iteration 0–12.3 %) for school surveys and 2.6 % (0–8.9 %) for community surveys. At any single site, the maximum difference in RDT prevalence over all survey visits was 7.6 %. Variation in both the prevalence of infection by RDT in school- and community-survey, as well as number of passively detected malaria cases at health facilities showed fluctuations during Phase 1, but no clear seasonal peak was observed (data not shown).

### Reported primary school enrolment of school-aged children

School-aged children comprised 32.7 % of the population living in sampled households, and 54.0 % of these children (range by site 42–62 %) were reported by their head of household to be enrolled at the local primary school. From multivariate modelling, key risk factors for non-enrolment of school-aged children in school were the distance of the household from school, low household wealth, and the number of children of school age in the household (Table 2). Odds of enrolment also varied with education level of the head of household, with children from households where the head had attended any education having higher odds of school enrolment than those from households headed by an individual with no education.

**Table 2:** Multivariate model of risk factors for non-enrolment of school-aged children (as reported by head of household during community survey)

	Odds ratio	95 % confidence interval	*P*
Age (increasing)	0.91	0.88, 0.95	<0.001
Number of children 7-16 years in household	1.20	1.08, 1.33	<0.001
Distance from school in km	1.57	1.30, 1.89	<0.001
Household wealth			
Poorest	1	-	-
Median	0.73	0.49, 1.10	0.132
Least poor	0.64	0.49, 0.84	0.001
Parental education			
None	1	-	-
Primary incomplete	0.66	0.51, 0.86	0.002
Primary complete or higher	0.64	0.42, 0.96	0.030

Fixed effects are presented, the multilevel model included random effects at household- and study-site level. Data were available from 1794 unique children and total 908 households, sampled from six sites in SNNPRS in 2012

### Child-reported reasons for absence from school

Across all sites, 94 % of children reported usually attending school five days per week. Of all absences reported by children, 28 % were due to illness, while 67 % of absences were in order to assist in the home or with farming activities. Variations by site were seen, with two sites reporting the majority of absences being due to illness. Where children reported absence from school due to illness, fever was the most common symptom (88 %); however, only 50 % of those who reported fever as a reason for absence from school attended a health facility.

### Phase 2 surveillance system pilot

Nine of the 10 cluster A schools submitted weekly summaries of the symptom and reported absence questionnaire. Schools collected the weekly indicators for a mean 11 weeks (range 6–14), overlapping with the peak transmission season from October to December. On average 68 children per school were interviewed each week (range 16–170). All ten cluster B schools submitted weekly estimates of absenteeism, calculated by summarising absent sessions recorded in attendance registers. Schools reported a mean 12 weeks of data (range 11–13). Schools summarised attendance for an average of 508 children each week (range 118 to 914).

In addition to the indicators collected from cluster A and B sites, available attendance registers were collected from a convenience sample of seven schools (five from cluster A and two from cluster B) for validation of weekly absenteeism in cluster B schools, and estimation of absenteeism in cluster A schools.

### Absenteeism and drop-out recorded by school attendance registers

Weekly summary rates of absenteeism calculated from attendance registers were similar between classes within schools (data not shown). However, changes in proportion of enrolled children who are absent from school is expected to increase over the semester due to drop-out. Strong evidence for an increase in average weekly absenteeism was found when analysing grade total absenteeism and allowing for clustering by school (*p* = 0.008). Absenteeism fluctuated on a weekly basis and varied by school, but showed an overall increase of 10 % during the study period.

### Do syndromic surveillance indicators from schools correlate with health facility malaria trends?

Testing of the syndromic surveillance system was hampered by the lack of strong seasonal increase in malaria cases seen at the study sites during both Phases 1 and 2. Health facility data from all sites demonstrated weekly fluctuations in number of cases, but no clear peak in transmission and no epidemic during either the minor or major transmission seasons (Phase 1 and 2, respectively).

**Fig. 3:**
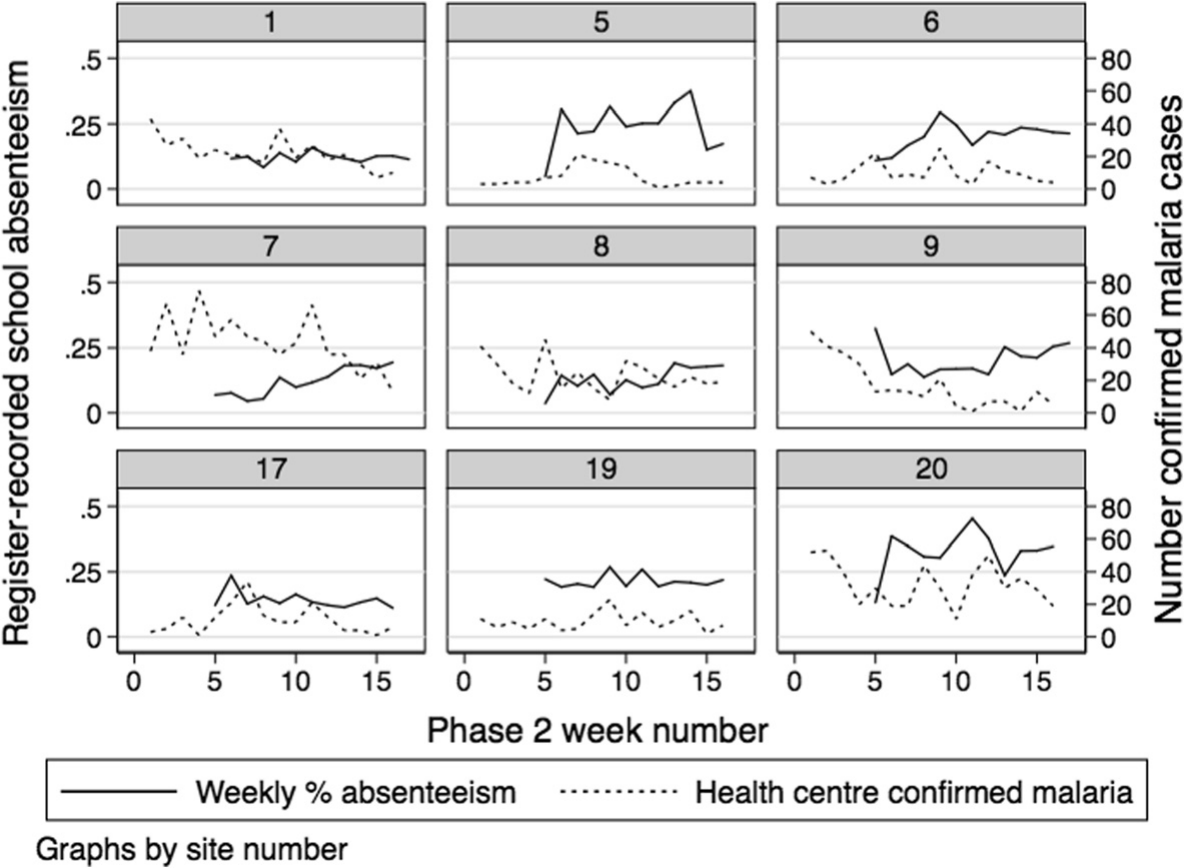
Phase 2 weekly proportion of children absent from school, calculated by school staff from attendance registers (solid line, primary y-axis) and total confirmed malaria infections identified at local health centre by routine passive surveillance (dotted line, secondary y-axis)

Few statistically significant correlations were seen between confirmed malaria at health facilities and the majority of the piloted indicators (i.e. child-reported fever, reported recent absence from school, absence from school due to illness, or teacher-summarised weekly absenteeism). There was evidence for an association between the proportion of child-days absent by week extracted from attendance registers collected for validation purposes and health facility total positive cases (*p* = 0.002) or RDT test positivity rate (*p* = 0.028). No evidence was found for an association between confirmed malaria at health facilities and teacher-summarised absenteeism from cluster B sites (*p* = 0.197, Fig. 3).

The difference in association with health facility data using absenteeism data from different sources may be a result of differences in malaria transmission levels between sites, with different locations contributing data to the attendance registers collected for validation purposes from both clusters A and B, and the teacher-summarised data from cluster B sites only. There were insufficient data to conduct site-specific analysis to further investigate patterns associations between piloted indicators and health facility data at different transmission levels.

## Discussion

This study is the first application of a surveillance system based on school absenteeism to the context of malaria epidemic early warning in a resource-poor setting. The study hypothesised that school attendance could act as a surrogate indicator of sudden increases in malaria burden or other infectious disease epidemics at community-level. However, during both the first and second phases of the study no seasonal peaks in malaria were observed from passive case detection at health facilities or cross-sectional survey RDT prevalence. Consequently, the majority of absences from school were reported to be not due to illness, and it was not possible to rigorously assess the study’s primary hypothesis.

In Phase 1 of the study, enrolment of school aged children was found to be lower than expected, although those who were enrolled attended routinely. At schools, collection of daily attendance occurs routinely, and schools were able to generate accurate weekly summary indicators to describe absenteeism. An alternative approach where teachers interview their pupils to answer simple questions on recent illness and absence was also completed but it was found to be less feasible for scale-up due to the time required for interviews. While this study demonstrated that school absenteeism is a feasible indicator for weekly collection and could supplement existing health system surveillance, this approach is likely to be less representative in locations with low primary school enrolment, particularly if there are common risk factors for non-enrolment and exposure to malaria.

### School enrolment

Our study found primary enrolment to be substantially lower than reported in national level data (42–62 % by site compared to 79 % national net enrolment in 2012 [24]). Our data concur with previous findings indicating that household wealth, education of household head and age of the child are associated with enrolment of children in primary school [50]. We found that the odds of enrolment were reduced with increasing number of school-aged children in the household, and increasing distance between household and school. Considering that universal enrolment and attendance at primary school has yet to be achieved in Ethiopia, it is likely that the sensitivity of a school-based syndromic surveillance system is reduced, since the whole community will not be captured at a school-level platform. Further piloting of school-based syndromic surveillance approaches is recommended in areas of higher primary school enrolment, where sensitivity will be improved.

### School absenteeism

Absence in schools can be classified as temporary or permanent (i.e. dropout). Illness was the second most commonly reported reason for temporary absence, but the most common reason was to assist with chores in the home or farm. Absence due to economic activities, attending market and lack of school materials were infrequently reported. Food insecurity has also been shown to be a determinant of school absenteeism and attainment in Ethiopia [51], and was a contributing factor to the severity of previous malaria epidemics in East Africa [20]. While the shift system used in many rural primary schools allows children to balance their schooling with farming, herding or domestic chores [52], it remains likely that school absenteeism will increase during periods of peak agricultural activity. Weekly absenteeism calculated from attendance registers was found to be similar across grades, and absenteeism could therefore be monitored from any grade as part of future implementation of the surveillance system.

While absence due to illness may be an intuitively more sensitive indicator for malaria surveillance, all-cause absenteeism is a more reliable indicator to collect on a daily basis. It is not feasible in this setting to trace a sibling, neighbour or parent to find out the reason for absence on their first missed day of school, unless a sibling attends the same class. It is not currently routine practice for schools in SNNPRS to record the reason for absence. This approach differs from school-based syndromic surveillance approaches in higher income countries, where reasons for absence can be rapidly determined by contacting parents by telephone.

The Ministry of Education target drop-out rate for the academic year 2010/11 of 8 %, but actual drop-out rate was 13 % [45]. Drop-out of approximately 10 % over the semester was observed at the study sites. While reasons for drop-out were not specifically investigated in the current study, drop-out can either be due to relatively stable factors such as number of children under five years in the household [53], as well as by economic shocks such as drought, crop failures, death and illness of family members [45].

### Benefits and drawbacks of the piloted system

Identification of valid indicators for piloting was limited by inclusion of only six sites in Phase 1 and the low malaria transmission experienced in SNNPRS in 2012. The study was also limited by inability to provide a formal sample size calculation for the comparisons we sought to undertake, due to lack of data describing changes in these indicators during normal and abnormal transmissions seasons.

Of the two piloted syndromic surveillance systems, monitoring school absenteeism is a less time-intensive activity than weekly completion of symptom questionnaires. Consequently, we propose absenteeism to be a more feasible indicator for long-term implementation. Absenteeism is routinely recorded and generation of a weekly absenteeism total is a simple addition to existing responsibilities for school staff. Registers in use were found to often not be standardised, with limited validation of class registers by senior staff since registers are often stored in teachers’ homes. For any future school absenteeism-based surveillance system, it is recommended to roll-out standard register formats and symbols for recording pupils’ daily presence and absence, as well as regular checking and feedback on attendance register completion by senior staff.

Differences in “normal” absenteeism rates between schools would likely remain due to systematic differences in populations across epidemic-prone areas. The usefulness of the system would be dependent on motivation of the school director and teachers to collect and assess absenteeism data, and report to health extension workers when increases occur. No thresholds would be assigned to schools, but it would be the responsibility of the school director to determine when absenteeism becomes unusual and to alert the health extension workers.

Syndromic surveillance systems in high-income countries generally use electronic data capture or web-based systems to collate reported and existing data for analysis [7, 8, 30, 31]. While low- and middle-income countries are increasingly adopting web-based or SMS technology, the current pilot did not require data to be reported upwards, and therefore these technology solutions were not required. Instead of submitting data to a central level for analysis and then response, the aim was to create simple indicators which would allow an alert to be passed from school to community health extension worker of possible increases in illness in the community. This information would then act as a prompt for the health extension worker to review their recent case data, or to conduct active surveillance in targeted areas of the kebele. While this system has low specificity, it builds upon existing links at community level between the school and health extension workers, through the kebele committee’s weekly meetings to discuss local issues, and may prove more sustainable in the long-term than any surveillance system requiring data to be reported upwards for analysis and feedback. The lack of strict thresholds to generate an alert from school data also allows flexibility to respond to rumours and local opinion, and could also capture non-malaria epidemics. Health extension workers routinely spend a proportion of their time making home visits in the kebele, and it is credible that intelligence from the syndromic surveillance system may allow targeting of home visits at the sub-kebele level to areas of highest absenteeism.

## Conclusions

In the current study, the lack of any malaria epidemic or strong seasonal peak in malaria transmission during the data collection period at the study sites prevented evaluation of the performance of a school-based syndromic surveillance system for malaria epidemic detection, resulting in inconclusive findings.

School-based surveillance approaches are limited by drop-out during the academic year and low rates of enrolment. Implementing a system without fixed thresholds for alert generation, and relying on subjective identification of “unusual” increases by school staff is one approach to avoid bias due to drop-out. Low school enrolment is a major limitation of the piloted surveillance system, and further investigation is required to assess if there are common risk factors for non-enrolment and malaria, and the extent to which this may reduce the sensitivity of school-based surveillance.

This syndromic surveillance approach could be further refined by piloting in malaria endemic or epidemic-risk settings with high primary school enrolment. Adapting the system to report absenteeism data by mobile phone to the local health facility or a central automated system combining syndromic and clinical data is an alternative approach to generate alerts. Mobile technology could also enable regular feedback to schools on the malaria situation in the wider area, or facilitate behaviour change communication messaging to pupils. Regardless of the data collation mechanism, piloting during a strong seasonal increase in malaria transmission or an epidemic would likely be required to demonstrate the sensitivity of a syndromic surveillance approach.

## References

[1] Abeku T, Van Oortmarssen GJ, Borsboom G, De Vlas SJ, Habbema JDF (2003). Spatial and temporal variations of malaria epidemic risk in Ethiopia: factors involved and implications. Acta Trop.

[2] Mutonga D, Langat D, Mwangi D, Tonui J, Njeru M, Abade A (2013). National surveillance data on the epidemiology of cholera in Kenya, 1997-2010. J Infect Dis.

[3] Halperin SA, Bettinger JA, Greenwood B, Harrison LH, Jelfs J, Ladhani SN (2012). The changing and dynamic epidemiology of meningococcal disease. Vaccine.

[4] Racloz V, Ramsey R, Tong S, Hu W (2012). Surveillance of dengue fever virus: a review of epidemiological models and early warning systems. PLoS Negl Trop Dis.

[5] Last JM (2001). A Dictionary of Epidemiology.

[6] Najera JA (1999). Prevention and control of malaria epidemics. Parassitologia.

[7] Kom Mogto CA, De Serres G, Douville Fradet M, Lebel G, Toutant S, Gilca R (2012). School absenteeism as an adjunct surveillance indicator: experience during the second wave of the 2009 H1N1 pandemic in Quebec, Canada. PLoS One.

[8] Kara EO, Elliot AJ, Bagnall H, Foord DG, Pnaiser R, Osman H (2012). Absenteeism in schools during the 2009 influenza A(H1N1) pandemic: a useful tool for early detection of influenza activity in the community?. Epidemiol Infect.

[9] Sasaki A, Hoen AG, Ozonoff A, Suzuki H, Tanabe N, Seki N (2009). Evidence-based tool for triggering school closures during influenza outbreaks, Japan. Emerg Infect Dis.

[10] Besculides M, Heffernan R, Mostashari F, Weiss D (2005). Evaluation of school absenteeism data for early outbreak detection, New York City. BMC Public Health.

[11] Yan W, Palm L, Lu X, Nie S, Xu B, Zhao Q (2013). ISS--an electronic syndromic surveillance system for infectious disease in rural China. PLoS One.

[12] Fan Y, Yang M, Jiang H, Wang Y, Yang W, Zhang Z (2014). Estimating the effectiveness of early control measures through school absenteeism surveillance in observed outbreaks at rural schools in Hubei, China. PLoS One.

[13] Cheng CKY, Channarith H, Cowling BJ (2013). Potential use of school absenteeism record for disease surveillance in developing countries, case study in Cambodia. PLoS One.

[14] Some ES (1994). Effects and control of highland malaria epidemic in Uasin Gishu District, Kenya. East Afr Med J.

[15] Fontaine RE, Najjar AE, Prince JS (1961). The 1958 malaria epidemic in Ethiopia. Am J Trop Med Hyg.

[16] President’s Malaria Initiative. Malaria Operational Plan for Ethiopia FY2015. In*.*; 2014.

[17] World Health Organization. Systems for the early detection of malaria epidemics in Africa. An analysis of current practices and future priorities. In*.*; 2006: 108.

[18] Federal Democratic Republic of Ethiopia MoH. Guidelines for malaria epidemic prevention and control in Ethiopia. 3rd edition: Addis Ababa, Ethiopia; 2009.

[19] Jima D, Wondabeku M, Alemu A, Teferra A, Awel N, Deressa W (2012). Analysis of malaria surveillance data in Ethiopia: what can be learned from the integrated disease surveillance and response system?. Malar J.

[20] Checchi F, Cox J, Balkan S, Tamrat A, Priotto G, Alberti KP (2006). Malaria epidemics and interventions, Kenya, Burundi, southern Sudan, and Ethiopia, 1999-2004. Emerg Infect Dis.

[21] Brown V, Abdir Issak M, Rossi M, Barboza P, Paugam A (1998). Epidemic of malaria in north-eastern Kenya. Lancet.

[22] Negash K, Kebede A, Medhin A, Argaw D, Babaniyi O, Guintran JO (2005). Malaria epidemics in the highlands of Ethiopia. East Afr Med J.

[23] Tanner M, Greenwood B, Whitty CJ, Ansah EK, Price RN, Dondorp AM (2015). Malaria eradication and elimination: views on how to translate a vision into reality. BMC Med.

[24] World Development Indicators: primary school enrollment [http://data.worldbank.org/indicator/SE.PRM.NENR] (2013). Accessed 30 Dec 2015.

[25] Stevenson JC, Stresman GH, Gitonga CW, Gillig J, Owaga C, Marube E (2013). Reliability of school surveys in estimating geographic variation in malaria transmission in the western Kenyan highlands. PLoS One.

[26] Baliraine FN, Afrane YA, Amenya DA, Bonizzoni M, Menge DM, Zhou G (2009). High prevalence of asymptomatic *Plasmodium falciparum* infections in a highland area of western Kenya: a cohort study. J Infect Dis.

[27] Ogutu B, Tiono AB, Makanga M, Premji Z, Gbadoe AD, Ubben D (2010). Treatment of asymptomatic carriers with artemether-lumefantrine: an opportunity to reduce the burden of malaria?. Malar J.

[28] Okell LC, Bousema T, Griffin JT, Ouedraogo AL, Ghani AC, Drakeley CJ (2012). Factors determining the occurrence of submicroscopic malaria infections and their relevance for control. Nat Commun.

[29] Henning KJ (2004). What is syndromic surveillance?. MMWR Morb Mortal Wkly Rep.

[30] Enserink R, Noel H, Friesema IH, de Jager CM, Kooistra-Smid AM, Kortbeek LM (2012). The KIzSS network, a sentinel surveillance system for infectious diseases in day care centers: study protocol. BMC Infect Dis.

[31] Andersson T, Bjelkmar P, Hulth A, Lindh J, Stenmark S, Widerstrom M. Syndromic surveillance for local outbreak detection and awareness: evaluating outbreak signals of acute gastroenteritis in telephone triage, web-based queries and over-the-counter pharmacy sales. Epidemiol Infect. 2013;142:303-13. 10.1017/S0950268813001088 PMC3891475 23672877

[32] Bounoure F, Beaudeau P, Mouly D, Skiba M, Lahiani-Skiba M (2011). Syndromic surveillance of acute gastroenteritis based on drug consumption. Epidemiol Infect.

[33] Patwardhan A, Bilkovski R (2012). Comparison: Flu prescription sales data from a retail pharmacy in the US with Google Flu trends and US ILINet (CDC) data as flu activity indicator. PLoS One.

[34] Signorini A, Segre AM, Polgreen PM (2011). The use of Twitter to track levels of disease activity and public concern in the U.S. during the influenza A H1N1 pandemic. PLoS One.

[35] Chunara R, Andrews JR, Brownstein JS (2012). Social and news media enable estimation of epidemiological patterns early in the 2010 Haitian cholera outbreak. Am J Trop Med Hyg.

[36] Broniatowski DA, Paul MJ, Dredze M (2013). National and local influenza surveillance through Twitter: An analysis of the 2012-2013 influenza epidemic. PLoS One.

[37] Mook P, Joseph C, Gates P, Phin N (2007). Pilot scheme for monitoring sickness absence in schools during the 2006/07 winter in England: can these data be used as a proxy for influenza activity?. Euro Surveill.

[38] Carme B, Sobesky M, Biard MH, Cotellon P, Aznar C, Fontanella JM (2003). Non-specific alert system for dengue epidemic outbreaks in areas of endemic malaria. A hospital-based evaluation in Cayenne (French Guiana). Epidemiol Infect.

[39] Kool JL, Paterson B, Pavlin BI, Durrheim D, Musto J, Kolbe A (2012). Pacific-wide simplified syndromic surveillance for early warning of outbreaks. Glob Public Health.

[40] Durrheim DN, Harris BN, Speare R, Billinghurst K (2001). The use of hospital-based nurses for the surveillance of potential disease outbreaks. Bull World Health Organ.

[41] John TJ, Rajappan K, Arjunan KK (2004). Communicable diseases monitored by disease surveillance in Kottayam district, Kerala state, India. Indian J Med Res.

[42] Randrianasolo L, Raoelina Y, Ratsitorahina M, Ravolomanana L, Andriamandimby S, Heraud JM (2010). Sentinel surveillance system for early outbreak detection in Madagascar. BMC Public Health.

[43] Yan WR, Nie SF, Xu B, Dong HJ, Palm L, Diwan VK (2012). Establishing a web-based integrated surveillance system for early detection of infectious disease epidemic in rural China: a field experimental study. BMC Med Inform Decis Mak.

[44] Federal Democratic Republic of Ethiopia MoH. Health Sector Development Program IV, 2010/11 - 2014/15. Final draft. In*.*; 2010.

[45] Woldehanna T, Hagos A. Shocks and Primary School Drop-out Rates: A Study of 20 Sentinel Sites in Ethiopia. In*.*: Young Lives, Oxford Department of International Development, Oxford University; 2012.

[46] Malaria Indicator Survey [http://www.malariasurveys.org/toolkit.cfm]. Accessed 30 Dec 2015.

[47] Ashton RA, Kefyalew T, Tesfaye G, Pullan RL, Yadeta D, Reithinger R (2011). School-based surveys of malaria in Oromia Regional State, Ethiopia: a rapid survey method for malaria in low transmission settings. Malar J.

[48] Gitonga CW, Karanja PN, Kihara J, Mwanje M, Juma E, Snow RW (2010). Implementing school malaria surveys in Kenya: towards a national surveillance system. Malar J.

[49] Filmer D, Pritchett LH (2001). Estimating wealth effects without expenditure data--or tears: an application to educational enrollments in states of India. Demography.

[50] Admassu KA. Primary school enrollment and dropout in Ethiopia: Household and school factors. In: Population Association of America 2011 Annual Meeting*.* Washington D.C, USA; 2011.

[51] Belachew T, Hadley C, Lindstrom D, Gebremariam A, Lachat C, Kolsteren P (2011). Food insecurity, school absenteeism and educational attainment of adolescents in Jimma Zone Southwest Ethiopia: a longitudinal study. Nutr J.

[52] Pereznieto P, Jones N. Young Lives Policy Brief 2. Educational Choices in Ethiopia: what determines whether poor children go to school? In: Young Lives*.* Oxford, UK; 2006: 12.

[53] Woldehanna T, Jones N, Tefera B. Children’s Educational Completion Rates and Achievement: Implications for Ethiopia's Second Poverty Reduction Strategy (2006-10). In: Young Lives Working Paper 18*.* Oxford: Young Lives; 2005.

[54] Rosewell A, Ropa B, Randall H, Dagina R, Hurim S, Bieb S (2013). Mobile phone-based syndromic surveillance system, Papua New Guinea. Emerg Infect Dis.

